# Gender Differences in Anxiety, Depression, and Nursing Needs Among Isolated Coronavirus Disease 2019 Patients

**DOI:** 10.3389/fpsyg.2021.615909

**Published:** 2021-06-07

**Authors:** Yifei Li, Juan Li, Zhen Yang, Jie Zhang, Lili Dong, Fusheng Wang, Jingping Zhang

**Affiliations:** ^1^Nursing Psychology Research Center, Xiangya School of Nursing, Central South University, Changsha, China; ^2^The Second Affiliated Hospital of Shantou University Medical College, Shantou, China

**Keywords:** coronavirus disease 2019, isolated patients, gender, nursing needs, hospital anxiety and depression scale, COVID-19

## Abstract

**Objective:**

This study explored gender differences in anxiety, depression, and nursing needs among isolated Coronavirus Disease 2019 (COVID-19) patients, with a particular focus on the influencing factors. The main goal was to elucidate breakthrough points and intervention targets for psychological counseling aimed at the promotion of overall health during isolation treatment.

**Methods:**

A survey was conducted to obtain information about the nursing needs of COVID-19 patients, with mental health assessed via the Hospital Anxiety and Depression Scale (HADS). Participants included 219 isolated COVID-19 patients at a Wuhan module hospital in Hubei province, China.

**Results:**

A total of 216 valid questionnaires were collected (98.63% retrieval rate). Of these participants, 21.76% had anxiety symptoms, while 17.59% had depression symptoms. Colleagues infected with COVID-19 (OR = 3.896, 95%CI: 1.555–9.764, *P* = 0.004) were the main influencing factors for anxiety symptoms, while marital status (OR = 2.700, 95% CI: 1.033–7.055, *P* = 0.043) and family members infected with COVID-19 (OR = 2.969, 95% CI: 1.243–7.095, *P* = 0.014) were the main influencing factors for depression symptoms. As for gender, male patients were generally more prone to depression and anxiety than female patients, especially those who were infected with colleagues. On the other hand, female patients reported greater concerns about safe treatment environments and communication with medical staff.

**Conclusion:**

This study found gender-based differences regarding the factors influencing anxiety and depression in isolated COVID-19 patients, with males reporting a greater general tendency for symptoms. On the other hand, female patients reported greater overall psychological nursing needs than males. Targeted nursing should thus be implemented to address specific psychological characteristics and nursing needs.

## Introduction

The highly contagious acute respiratory infection leading to the illness known as Coronavirus Disease 2019 (COVID-19) began spreading across the globe in late 2019 (Wang C. et al., 2020). It has now infected more than 149 million people, resulting in 3.15 million total deaths as of April 30, 2021 (World Health Organization (WHO), 2021). COVID-19 is thus referred to as the sixth worldwide public health crisis (Arab-Mazar et al., 2020). According to the existing case data, COVID-19 is mainly manifested by fever, dry cough and fatigue, and a small number of patients are accompanied by upper respiratory tract and digestive tract symptoms such as nasal obstruction, runny nose and diarrhea. About half of the patients develop dyspnea 1 week later, which in severe cases can lead to acute respiratory distress syndrome or septic shock and even death (Chen and Li, 2020). The primary routes of transmission of the COVID-19 are through respiratory droplets and close person-to-person contact (Amirian, 2020).

In this context, quarantine is one of the most effective measures for halting the spread of the virus (Chen Z. et al., 2020). In fact, the diagnosis and treatment plan issued by the National Health Commission of the People’s Republic of China also emphasizes that COVID-19 patients must be isolated and treated in designated hospitals (National Health Commission of the People’s Republic of China, 2020). However, this practice has created a dilemma. While isolation provides epidemiological benefits, it can also cause psychosocial harm, thereby resulting in more negative emotions and nursing needs among affected patients (Sharma et al., 2020).

A previous study on severe acute respiratory syndrome (SARS) patients who were placed in isolation found that 96.6% thus experienced feelings of inferiority, loneliness, and abandonment (Yang, 2004). A later follow-up study on discharged SARS patients found that more than one-third had incurred psychological problems, including post-traumatic stress disorder, anxiety, and depression (Kwek et al., 2006). Similarly, an investigation among isolated COVID-19 patients found that anxiety and depression were common psychological problems, with respective incidence rates of 38.5 and 35.9% (Nie et al., 2020). These negative psychological impacts not only harm mental health, but may also affect disease recovery (Fu and Luo, 2011; Pang, 2016).

One of the important purposes of human activity is to satisfy needs. In this regard, isolated COVID-19 patients may require greater nursing needs due to several unique conditions, including separation from relatives, fear of the disease, and unfamiliarity induced by isolation (Fan et al., 2020). Wang Z. Y. et al. (2020) found that COVID-19 patients generally experienced anxiety and depression when being admitted to the hospital. However, most of these symptoms are alleviated through continued treatment and satisfaction with nursing services (Wang Z. Y. et al., 2020). In other words, medical staff can more efficiently deal with negative emotions through a better understanding of the specific nursing needs, thus improving overall mental health for patients.

Many previous studies on COVID-19 patients have found gender-based differences in both anxiety and depression. For example, female patients are more likely to experience negative emotions related to anxiety and depression than male patients (Gu et al., 2020; Nie et al., 2020). Similar studies on SARS patients have also found that males and females tend to have different nursing needs, with female patients reporting a greater need for contact with relatives (Li et al., 2003). These types of studies can produce valuable information for medical staff during targeted interventions aimed at reducing negative emotions for patients. However, there is still a lack of information on gender-based differences in anxiety, depression, and nursing needs among COVID-19 patients. As such, this survey study investigated differences in sociodemographic characteristics, anxiety, depression, and nursing needs between male and female COVID-19 patients who were placed in isolation. Our findings provide a basis for interventions and services aimed at alleviating negative emotions while promoting overall mental health.

## Materials and Methods

This study implemented a cross-sectional design in which an online survey was conducted to assess anxiety, depression, and nursing needs among isolated COVID-19 patients at a Wuhan module hospital in Hubei province, China. All participants admitted to the hospital were symptomatic patients with mild COVID-19. The data collection period lasted from March 3–7, 2020.

### Participants

Patients with COVID-19 were invited to participate in the online survey through the Wenjuanxing platform. All questionnaires were completed anonymously. Specific inclusion criteria were set as follows: patients who (1) were diagnosed with COVID-19 according to treatment protocols, (2) allowed to voluntarily participate in this study, (3) provided their informed written consent. Exclusion criteria were set as follows: patients with self-reported histories of neurological disorders, mental illness, and/or other serious systemic disorders. This resulted in a total of 219 eligible participants patients. After removing data from those with incomplete questionnaires, 216 were ultimately included in the analyses. This study was reviewed by the Institutional Review Board at the researchers’ university (Ethical Grant Number: E202073). All patients provided online informed consent prior to their participation.

### Measurements

#### Demographic Characteristics

Demographic data were collected using a self-designed questionnaire, with reported characteristics including gender, age, marital status, education level, occupation, living area, monthly family income, hospitalization time, and whether any family members or friends were infected with COVID-19. Other questions included the following: “Do you have any symptoms such as fever, cough, sore throat, chest tightness, diarrhea or fatigue?” and “Have you felt any improvement since admission?”.

#### Hospital Anxiety and Depression Scale

Participants also completed the Hospital Anxiety and Depression Scale (HADS) (Zigmond and Snaith, 1983), which was used to determine the presence of depression and anxiety. The HADS consists of 14 items, including seven for depression and seven for anxiety. Scores for each subscale may range from 0–21, with scores of 0–7 denoting no symptoms, 8–10 denoting borderline abnormal cases, and 11–21 denoting abnormal cases, respectively. Higher scores on each subscale reflect more severe symptoms. In this study, Cronbach’s alpha values of 0.842 and 0.850 were achieved for the anxiety and depression subscales, respectively.

#### Questionnaire on the Nursing Needs of COVID-19 Patients

Referring to the evaluation model of “functional health patterns” established by Gordon (Sheng et al., 2003) and based on expert advice, we reviewed relevant literature (Zhang, 2012) and designed the “Questionnaire on the nursing Needs of COVID-19 patients” (Table 4). The scale consists of 18 items that are each answered with one of three options, including “need,” “does not matter,” and “do not need.” In this study, the scale achieved a Cronbach’s alpha value of 0.820.

### Data Analysis

IBM SPSS Version 21.0 was used for all statistical analyses (significance threshold set at 0.05). Continuous descriptive data were expressed as means and standard deviations (SDs), whereas categorical data were expressed as frequencies and percentages via the chi-squared test. A multivariate logistic regression was conducted to investigate potential influencing factors for anxiety and depression in three groups, including all participants, male participants, and female participants. The associations between anxiety, depression, and influencing factors were presented as odds ratios (ORs) and 95% confidence intervals (CIs).

## Results

### Analyzing Basic Participant Characteristics

This study analyzed data from 216 isolated COVID-19 patients, including 124 males and 92 females (average age of 39.21 ± 9.91; range of 18–64 years). The length of hospital stay ranged from 1–38 days, with an average of 13.51 ± 4.17. Of all participants, 21.76% had anxiety symptoms, while 17.59% had depression symptoms. Colleagues infected with COVID-19 were associated with anxiety (χ*2* = 7.446, *P* = 0.006), while family members infected with COVID-19 were associated with depression (χ*2* = 4.743, *P* = 0.029). Table 1 shows the relationship between basic characteristics and the presence of depression and anxiety symptoms.

**TABLE 1:** Demographic characteristics of patients (*N* = 216).

Variables		Non-anxiety, *N* (%)	Anxiety, *N* (%)	χ*2*	*P*	Non-depression, *N* (%)	Depression, *N* (%)	χ*2*	*P*
Gender	Male	102 (47.22)	22 (10.19)	2.760	0.097	102 (47.22)	22 (10.19)	0.004	0.947
	Female	67 (31.02)	25 (11.57)			76 (35.18)	16 (7.41)		
Age, year	18–39	93 (43.05)	22 (10.19)	3.294	0.193	92 (42.59)	23 (10.65)	1.954	0.377
	40–59	70 (32.41)	25 (11.57)			80 (37.04)	15 (6.94)		
	>60	6 (2.78)	0			6 (2.78)	0		
Marital status	Unmarried	34 (15.74)	7 (3.24)	1.654	0.647	37 (17.13)	4 (1.85)	3.473	0.324
	Married	129 (59.72)	38 (17.59)			135 (62.50)	32 (14.82)		
	Divorce	4 (1.85)	2 (0.93)			5 (2.32)	1 (0.46)		
	Bereft of one’s spouse	2 (0.93)	0			1 (0.46)	1 (0.46)		
Educational level	Primary school or below	3 (1.39)	0	3.153	0.533	1 (0.46)	2 (0.93)	5.289	0.259
	Junior high school	19 (8.79)	9 (4.17)			23 (10.64)	5 (2.32)		
	High school	39 (18.05)	9 (4.17)			41 (18.98)	7 (3.24)		
	Junior college or bachelor	100 (46.29)	26 (12.04)			104 (48.15)	22 (10.18)		
	Master or above	9 (4.17)	2 (0.93)			9 (4.17)	2 (0.93)		
Occupation	Students	7 (3.24)	0	7.011	0.220	6 (2.78)	1 (0.46)	4.081	0.538
	HCWs	2 (0.93)	0			2 (0.93)	0		
	Government employees or institutional employees	82 (37.96)	18 (8.33)			87 (40.28)	13 (6.02)		
	Freelancers	28 (12.96)	13 (6.02)			33 (15.28)	8 (3.70)		
	Unemployed	8 (3.70)	1 (0.46)			7 (3.24)	2 (0.93)		
	Others	42 (19.45)	15 (6.95)			43 (19.90)	14 (6.48)		
Living area	Urban	161 (74.54)	46 (21.30)	0.625	0.429	169 (78.24)	38 (17.59)	2.005	0.157
	Rural	8 (3.70)	1 (0.46)			9 (4.17)	0		
Monthly Family income, ¥^a^	2000<	6 (2.78)	2 (0.93)	4.284	0.232	6 (2.78)	2 (0.93)	0.958	0.811
	2001–4000	28 (12.96)	14 (6.48)			33 (15.27)	9 (4.17)		
	4001–6000	43 (19.91)	10 (4.63)			44 (20.37)	9 (4.17)		
	>6000	92 (42.59)	21 (9.72)			95 (43.98)	18 (8.33)		
Hospitalization time, day	0–7	14 (6.48)	4 (1.85)	1.441	0.696	16 (7.40)	2 (0.93)	1.695	0.638
	8–14	66 (30.55)	22 (10.19)			74 (34.26)	14 (6.48)		
	15–21	87 (40.28)	21 (9.72)			86 (39.81)	22 (10.19)		
	>22	2 (0.93)	0			2 (0.93)	0		
Are your family members infected with COVID-19?	No	67 (31.02)	18 (8.33)	0.028	0.867	76 (35.19)	9 (4.17)	4.743	0.029^∗^
	Yes	102 (47.22)	29 (13.43)			102 (47.22)	29 (13.42)		
Are your friends infected with COVID-19?	No	154 (71.30)	43 (19.91)	0.006	0.938	165 (76.39)	32 (14.81)	2.811	0.094
	Yes	15 (6.94)	4 (1.85)			13 (6.02)	6 (2.78)		
Are your colleagues infected with COVID-19?	No	147 (68.05)	33 (15.28)	7.446	0.006^∗^	151 (69.91)	29 (13.42)	1.635	0.201
	Yes	22 (10.19)	14 (6.48)			27 (12.50)	9 (4.17)		
Are your neighbors infected with COVID-19?	No	152 (70.37)	42 (19.44)	0.013	0.908	159 (73.61)	35 (16.20)	0.264	0.607
	Yes	17 (7.87)	5 (2.32)			19 (8.80)	3 (1.39)		
Do you have any symptoms such as fever, cough, sore throat, chest tightness, diarrhea or fatigue?	No	71 (32.87)	17 (7.87)	0.520	0.471	76 (35.18)	12 (5.56)	1.603	0.205
	Yes	98 (45.37)	30 (13.89)			102 (47.22)	26 (12.04)		
Have you felt any improvement since admission?	Improved significantly	97 (44.91)	21 (9.72)	2.536	0.469	98 (45.37)	20 (9.26)	0.281	0.964
	Slight improvement	43 (19.91)	16 (7.41)			49 (22.69)	10 (4.63)		
	unchanged	25 (11.57)	9 (4.17)			27 (12.50)	7 (3.24)		
	Slight worse	4 (1.85)	1 (0.46)			4 (1.85)	1 (0.46)		
	Significantly worse								

^a^As of November 18, 2020, 1 ¥ = $0.15 US. *P < 0.05 N, Number; HCWs, health care workers; COVID-19, coronavirus disease 2019.

### Gender-Focused Multivariate Analysis of Anxiety Symptoms

A multivariate logistic regression analysis showed that colleagues infected with COVID-19 were the main influencing factors for anxiety symptoms (OR = 3.896, 95% CI: 1.555–9.764, *P* = 0.004), particularly among male participants (OR = 13.286, 95% CI: 2.902–60.832, *P* = 0.001). For female participants, on the other hand, occupation (OR = 1.393, 95% CI: 1.007–1.926, *P* = 0.045) and the item “Do you have any symptoms such as fever, cough, sore throat, chest tightness, diarrhea or fatigue?” (OR = 0.255, 95% CI: 0.068–0.959, *P* = 0.043) were the main influencing factors of anxiety symptoms (Table 2).

**TABLE 2:** Gender-influencing factors of anxiety symptoms in isolated COVID-19 patients.

Variables		All (*N* = 216)				Male (*N* = 124)				Female (*N* = 92)			
		*B*	OR	95%CI	*P*	*B*	OR	95%CI	*P*	*B*	OR	95%CI	*P*
Anxiety	Gender	0.499	1.646	0.806–3.364	0.171								
	Age	0.008	1.008	0.963–1.056	0.725	−0.016	0.984	0.916–1.057	0.652	0.023	1.024	0.947–1.106	0.558
	Marital status	0.095	1.100	0.452–2.675	0.834	0.736	2.088	0.442–9.873	0.353	−1.245	0.288	0.057–1.464	0.134
	Educational level	0.291	1.337	0.777–2.302	0.294	0.087	1.091	0.479–2.486	0.836	0.450	1.569	0.624–3.942	0.338
	Occupation	0.198	1.219	0.992–1.497	0.060	0.186	1.205	0.877–1.656	0.250	0.331	1.393	1.007–1.926	0.045^∗^
	Living area	−1.359	0.257	0.026–2.524	0.244	−19.778	0.000	0.000–0.000	0.999	0.758	2.134	0.093–48.854	0.635
	Monthly Family income	−0.390	0.677	0.430–1.067	0.093	−0.406	0.666	0.339–1.310	0.239	−0.182	0.833	0.393–1.768	0.635
	Hospitalization time	−0.027	0.974	0.889–1.067	0.567	−0.102	0.903	0.780–1.045	0.171	0.109	1.115	0.925–1.345	0.253
	Are your family members infected with COVID-19?	0.312	1.366	0.653–2.853	0.407	0.963	2.619	0.740–9.269	0.135	−0.403	0.668	0.218–2.052	0.481
	Are your friends infected with COVID-19?	−0.890	0.411	0.099–1.698	0.219	−0.390	0.677	0.075–6.079	0.727	−1.428	0.240	0.028–2.033	0.190
	Are your colleagues infected with COVID-19?	1.360	3.896	1.555–9.764	0.004^∗^	2.587	13.286	2.902–60.832	0.001^∗^	0.298	1.348	0.332–5.478	0.677
	Are your neighbors infected with COVID-19?	−0.215	0.806	0.235–2.770	0.732	−1.435	0.238	0.020–2.868	0.258	1.038	2.825	0.452–17.674	0.267
	Do you have any symptoms such as fever, cough, sore throat, chest tightness, diarrhea or fatigue?	−0.279	0.757	0.362–1.583	0.459	0.116	1.124	0.386–3.273	0.831	−1.368	0.255	0.068–0.959	0.043^∗^
	Have you felt any improvement since admission?	0.225	1.253	0.818–1.918	0.300	−0.088	0.916	0.481–1.743	0.789	0.353	1.423	0.682–2.969	0.348

*P < 0.05. N, Number; CI, confidence interval; OR, odds ratio; COVID-19, coronavirus disease 2019.

### Gender-Focused Multivariate Analysis of Depression Symptoms

A multivariate logistic regression analysis showed that marital status (OR = 2.700, 95% CI: 1.033–7.055, *P* = 0.043) and family members infected with COVID-19 (OR = 2.969, 95%CI: 1.243–7.095, *P* = 0.014) were the main influencing factors for depression symptoms. For male participants, the main factors included age (OR = 0.859, 95% CI: 0.767–0.962, *P* = 0.009), marital status (OR = 30.409, 95%CI: 3.485–265.350, *P* = 0.002), monthly family income (OR = 0.378, 95% CI: 0.165–0.867, *P* = 0.022), family members infected with COVID-19 (OR = 19.903, 95%CI: 2.616–151.430, *P* = 0.004), and colleagues infected with COVID-19 (OR = 21.639, 95%CI: 2.571–182.102, *P* = 0.005). However, none of the tested factors showed statistically significant influences on depression symptoms among female participants (Table 3).

**TABLE 3:** Gender-influencing factors of depression symptoms in isolated COVID-19 patients.

Variables		All (*N* = 216)				Male (*N* = 124)				Female (*N* = 92)			
		*B*	OR	95%CI	*P*	*B*	OR	95%CI	*P*	*B*	OR	95%CI	*P*
Depression	Gender	−0.178	0.837	0.378–1.855	0.661								
	Age	−0.032	0.968	0.919–1.020	0.220	−0.152	0.859	0.767–0.962	0.009^∗^	0.023	1.023	0.939–1.114	0.604
	Marital status	0.993	2.700	1.033–7.055	0.043^∗^	3.415	30.409	3.485–265.350	0.002^∗^	−0.225	0.798	0.175–3.636	0.771
	Educational level	−0.011	0.989	0.536–1.825	0.972	0.813	2.256	0.734–6.934	0.156	−0.474	0.623	0.236–1.641	0.338
	Occupation	0.205	1.227	0.979–1.539	0.075	0.391	1.479	0.985–2.221	0.059	0.196	1.216	0.849–1.742	0.285
	Living area	−18.603	0.000	0.000–0.000	0.999	−17.949	0.000	0.000–0.000	0.999	−19.731	0.000	0.000–0.000	0.999
	Monthly Family income	−0.228	0.797	0.483–1.313	0.373	−0.973	0.378	0.165–0.867	0.022^∗^	0.511	1.667	0.701–3.966	0.248
	Hospitalization time	0.046	1.047	0.948–1.156	0.368	0.226	1.253	0.992–1.583	0.058	−0.023	0.978	0.812–1.177	0.812
	Are your family members infected with COVID-19?	1.088	2.969	1.243–7.095	0.014^∗^	2.991	19.903	2.616–151.430	0.004^∗^	0.419	1.520	0.434–5.319	0.513
	Are your friends infected with COVID-19?	0.732	2.080	0.556–7.782	0.277	1.154	3.170	0.228–44.027	0.390	0.463	1.589	0.228–11.076	0.640
	Are your colleagues infected with COVID-19?	0.932	2.539	0.890–7.244	0.081	3.075	21.639	2.571–182.102	0.005^∗^	−0.661	0.516	0.092–2.903	0.453
	Are your neighbors infected with COVID-19?	−1.515	0.220	0.043–1.135	0.070	−26.017	0.000	0.000–0.000	0.998	0.341	1.406	0.197–10.058	0.734
	Do you have any symptoms such as fever, cough, sore throat, chest tightness, diarrhea or fatigue?	−0.528	0.590	0.263–1.319	0.198	−0.771	0.463	0.126–1.706	0.247	−1.181	0.307	0.069–1.366	0.121
	Have you felt any improvement since admission?	0.133	1.142	0.710–1.838	0.584	0.263	1.301	0.570–2.969	0.532	0.470	1.600	0.660–3.875	0.298

*P < 0.05. N, Number; CI, confidence interval; OR, odds ratio; COVID-19, coronavirus disease 2019.

### Gender-Based Nursing Needs

As shown in Table 4, timely examinations and treatments, understanding of the disease prognosis, understanding the effects and side effects of therapeutic agents, and guidance for preventing recurrence were more than 95% in both male and female participants. However, female participants reported a greater general need for three items, including being cared for by family members, safe and comfortable treatment environments, and communication with doctors and nurses. These differences were statistically significant (*P* < 0.05).

**TABLE 4:** Gender-nursing needs of isolated COVID-19 patients (*N* = 216).

Variables	Male						Female						χ*^2^*
		
	Need		Does not matter		Do not need		Need		Does not matter		Do not need		
	*n*	%	*n*	%	*n*	%	*n*	%	*n*	%	*n*	%	
Be understood and sympathized.	70	56.45	18	14.52	36	29.03	44	47.83	14	15.22	34	36.95	0.410
Being taken care of by family members.	33	26.61	17	13.71	74	59.68	33	35.87	4	4.35	55	59.78	0.044*
Provide for oneself life.	40	32.26	15	12.10	69	55.64	33	35.87	10	10.87	49	53.26	0.849
Keep in touch with colleagues or friends.	81	65.32	25	20.16	18	14.52	59	64.13	21	22.83	12	13.04	0.874
Get the attention of the society.	63	50.81	29	23.39	32	25.80	50	54.35	13	14.13	29	31.52	0.216
You will receive a warm reception during the treatment.	103	83.06	14	11.29	7	5.65	78	84.78	12	13.04	2	2.18	0.432
The treatment environment is safe and comfortable.	104	83.87	15	12.10	5	4.03	87	94.57	5	5.43	0	0	0.012*
Timely examination and treatment.	118	95.16	5	4.03	1	0.81	90	97.82	1	1.09	1	1.09	0.420
Communicate with your doctor or nurse.	116	93.55	8	6.45	0	0	90	97.82	1	1.09	1	1.09	0.047*
When you are in hospital, you can confide in others if you have unpleasant things.	91	73.39	25	20.16	8	6.45	75	81.53	12	13.04	5	5.43	0.349
Communicate and talk with fellow patients.	91	73.39	24	19.35	9	7.26	73	79.35	15	16.30	4	4.35	0.532
Doctor or nurse can tell the truth about a patient’s condition.	115	92.74	8	6.45	1	0.81	87	94.56	4	4.35	1	1.09	0.785
Learn about the spread of COVID-19.	112	90.32	11	8.87	1	0.81	86	93.48	6	6.52	0	0	0.557
Understand the effects and side effects of therapeutic agents.	121	97.58	3	2.42	0	0	89	96.74	3	3.26	0	0	0.510
Understand the prognosis of the disease	120	96.77	4	3.23	0	0	91	98.91	1	1.09	0	0	0.291
Guidance for preventing recurrence.	119	95.97	5	4.03	0	0	90	97.82	2	2.18	0	0	0.362
Understand hospital and medical team structure.	93	75.00	22	17.74	9	7.26	66	71.74	21	22.83	5	5.43	0.597
Know the names of doctors and nurses.	79	63.71	34	27.42	11	8.87	59	64.13	27	29.35	6	6.52	0.802

*P < 0.05.

## Discussion

### Analyzing Anxiety, Depression, and the Influencing Factors for Both

As mentioned, 21.76% of participants had anxiety symptoms, while 17.59% had depression symptoms. These results may partly be related to the fact that all were considered mild patients with generally good prognoses and low mortality risks (Xiong et al., 2020). The average length of stay was 13.51 ± 4.17 days. In this context, familiarity with both the inpatient environment and medical staff may have reduced anxiety and depression during the isolation period (Wang Z. Y. et al., 2020). After receiving a series of treatments, 54.63% believed that their condition had significantly improved, which was also conducive to the reduction of negative emotions related to general health concerns.

Participants whose colleagues were also infected with COVID-19 were more prone to anxiety symptoms. This may be related to COVID-19 is highly contagious. Colleague infections mean that the workplace of participants may be in the outbreak area, which could company bankruptcy or patient unemployment. Male participants as pillars of their family, are particularly more likely to experience anxiety symptoms due to these stresses (Shi et al., 2020). On the other hand, occupation and COVID-19 accompanying symptoms can affect the anxiety symptoms in female participants. Female participants who were unemployed or freelance workers and who developed symptoms related to COVID-19 had higher anxiety. This may be related to the influence of job instability on their economic conditions (Xiao et al., 2020) and/or physical discomfort caused by the disease (Xue et al., 2008). More specifically, previous studies have shown that hypoxia and dyspnea are among the most common symptoms for COVID-19 patients (Chen N. et al., 2020). In this context, dyspnea is significantly and positively correlated with negative emotions such as anxiety, depression, and fear (Jiao, 2013).

Previous studies on depression in COVID-19 patients have found that those who are married or have other family members with the disease are more likely to develop depression symptoms. In particular, married men aged 18–39 years with lower family incomes and family members or colleagues with COVID-19 are more likely to suffer from depression (Shi et al., 2020; Xiao et al., 2020). In this regard, individuals with those attributes are also more likely undertake heavy work tasks and family support roles, thus incurring greater psychological burdens and negative emotions (Xiao et al., 2020). Male isolated COVID-19 patients with lower monthly household incomes may experience even more depressive symptoms due to financial stress (Cheng et al., 2020), while those with infected family members and colleagues may develop additional concerns. In general, disease-related uncertainties may lead to depression during isolation, particularly when there is otherwise a high amount of close daily contact with family members and/or colleagues. In other words, the isolation environment may create a lack of information, thus increasing the level of concern.

The influencing factors for anxiety and depression differ between genders. This may be related to the different roles, responsibilities, and jobs held by men and women in society (Weich et al., 1998). Further, research has shown that women are more prone to negative emotions when facing stressful events, particularly when under the influences of physiological and cognitive factors (McLean and Hope, 2010). When treating COVID-19 patients, medical staff should therefore implement targeted psychological interventions aimed at the different psychological characteristics of male and female patients. This will provide a better way to target the unique elements that contribute to anxiety and depression while enhancing confidence and courage among those facing the disease.

### Analyzing Nursing Needs

In this study, more than 95% of patients reported on timely examinations and treatments, an understanding of the disease prognosis, understanding the effects and side effects of therapeutic agents, and guidance for preventing recurrence. This may be related to the nature and severity of COVID-19 itself. Indeed, novel coronaviruses may pose serious harm to humans, and are typically highly contagious (Wei and Li, 2020). For COVID-19, the number of deaths in China now exceeds the total number during the SARS epidemic of 2002–2003 (Jingwei Network, 2003), and there is no specific drug that targets the virus. Patients may therefore express more nursing needs related to examinations, drug treatments, prognoses, and recurrence and prevention guidance due to increased worries and uncertainties about their conditions and outlooks.

Notably, female participants reported greater need than male participants in three specific areas, including being cared for by family members, a safe and comfortable treatment environment, and communication with doctors and nurses. These differences were statistically significant, which is consistent with previous research (Li et al., 2003). In general, this may be due to the fact that female patients are more sensitive to the perception of negative emotions and more likely to have physiological/psychological reactions related to negative emotions than males (McLean and Anderson, 2009; Zhang and Li, 2020). Healthcare workers should therefore pay increased attention to the unique psychological needs of patients while increasing the overall level of communication. This includes a particular focus on providing timely diagnosis, treatment, and nursing information to female patients.

## Limitations

This study had two main limitations. First, it only investigated COVID-19 patients at one makeshift hospital in Wuhan, which may have resulted in selection bias. Second, only general data from patients were analyzed. In this case, the effects of personality traits, disease cognition, social support, and other factors are unknown. Future studies should address both these issues, thus contributing to a more comprehensive discussion on the psychological status of COVID-19 patients.

## Conclusion

Of the isolated COVID-19 patients included in this analysis, 21.76% had anxiety symptoms, while 17.59% had depression symptoms. When broken down to look at gender, there were different influencing factors for both anxiety and depression. More specifically, female patients reported greater psychological nursing needs than male patients. While treating the illness, healthcare workers should therefore pay increased attention to any emotional changes, especially among patients who are more susceptible to anxiety and depression. Targeted nursing should thus be implemented to meet specific nursing needs, while psychological interventions should aim to promote overall mental health while preventing the development of more serious mental diseases.

## Data Availability Statement

The raw data supporting the conclusions of this article will be made available by the authors, without undue reservation.

## Ethics Statement

The studies involving human participants were reviewed and approved by the Xiang Ya Nursing School of Central South University (Ethical Grant Number: E201946). – Written informed consent to participate in this study was provided by the patient/participants.

## Author Contributions

JPZ was the primary investigator of the study and provided comments and ideas, and revised this manuscript. YL conducted the data analysis and contributed to writing the manuscript. JL and JZ helped with the data analysis and coding and contributed to writing the manuscript. LD and FW helped with the questionnaire survey. ZY contributed conceptually to data generation and analysis and suggested revisions. All authors contributed to the article and approved the submitted version.

## Conflict of Interest

The authors declare that the research was conducted in the absence of any commercial or financial relationships that could be construed as a potential conflict of interest.

## References

[B1] Amirian E. S. (2020). Potential fecal transmission of SARS-CoV-2: current evidence and implications for public health. *Int. J. Infec. Dis.* 95 363–370. 10.1016/j.ijid.2020.04.057 32335340 PMC7195510

[B2] Arab-Mazar Z. Sah R. Rabaan A. A. Dhama K. Rodriguez-Morales A. J. (2020). Mapping the incidence of the COVID-19 hotspot in Iran - implications for travellers. *Travel Med. Infect. Dis.* 34:101630. 10.1016/j.tmaid.2020.101630 32184130 PMC7118655

[B3] Chen N. Zhou M. Dong X. Qu J. Gong F. Han Y. (2020). Epidemiological and clinical characteristics of 99 cases of 2019 novel coronavirus pneumonia in Wuhan, China: a descriptive study. *Lancet* 395 507–513. 10.1016/S0140-6736(20)30211-7 32007143 PMC7135076

[B4] Chen Y. H. Li W. (2020). The Clinical Symptoms, Classification and Diagnosis of COVID-19. *Genomics Appl. Biol.* 39 3904–3907. 10.13417/j.gab.039.003904

[B5] Chen Z. Zhuang Y. J. Li J. Yang X. L. Li J. Feng Y. (2020). Medical management of hospitals designated for patients with COVID-19. *Chin. J. Nosocomiol.* 30 821–825. 10.11816/cn.ni.2020-200237

[B6] Cheng J. G. Tan X. D. Zhang L. Zhu S. R. Yao H. Liu B. (2020). Research on the psychological status and influencing factors of novel coronavirus pneumonia patients and people under medical observation. *J. Nurs. Adm.* 20 247–251.

[B7] Fan P. Aloweni F. Lim S. H. Ang S. Y. Perera K. Quek A. H. (2020). Needs and concerns of patients in isolation care units - learnings from COVID-19: a reflection. *World J. Clin. Cases* 8 1763–1766. 10.12998/wjcc.v8.i10.1763 32518768 PMC7262715

[B8] Fu Y. N. Luo X. (2011). The role of psychosocial factors in the significant emergencies disease responding. *Chin. J. Soc. Med.* 28 279–280.

[B9] Gu Y. Zhu Y. Xu F. Xi J. Xu G. (2020). Factors associated with mental health outcomes among patients with COVID-19 treated in the Fangcang shelter hospital in China. *Asia Pacific Psychiatry* 13:e12443. 10.1111/appy.12443 33135397

[B10] Jiao T. (2013). *A Prevalence of Anxiety and Depression in Patients With an Acute Exacerbation of COPD.* Hefei: Anhui Medical University.

[B11] Jingwei Network (2003). *The Ministry of Health Announced the Last Daily Outbreak: No SARS Patients in Mainland China.* Avaliable online at: http://www.huaxia.com/xw/dlxw/2003/08/234782.html (accessed August 17, 2003).

[B12] Kwek S. K. Chew W. M. Ong K. C. Ng A. W. Lee L. S. Kaw G. (2006). Quality of life and psychological status in survivors of severe acute respiratory syndrome at 3 months postdischarge. *J. Psychosom. Res.* 60 513–519. 10.1016/j.jpsychores.2005.08.020 16650592 PMC7094294

[B13] Li S. J. Yang S. Zhang Y. R. Zhang Y. Wang Y. L. Sun C. L. (2003). Investigation of SARS patients’ health service needs. *Chin. J. Nurs.* 12 18–20.

[B14] McLean C. P. Anderson E. R. (2009). Brave men and timid women? A review of the gender differences in fear and anxiety. *Clin. Psychol. Rev.* 29 496–505. 10.1016/j.cpr.2009.05.003 19541399

[B15] McLean C. P. Hope D. A. (2010). Subjective anxiety and behavioral avoidance: gender, gender role, and perceived confirmability of self-report. *J. Anxiety Disord.* 24 494–502. 10.1016/j.janxdis.2010.03.006 20381303

[B16] National Health Commission of the People’s Republic of China (2020). *Interpretation of COVID-19 Diagnosis and Treatment Protocol (trial version 7).* Avaliable online at: http://www.nhc.gov.cn/yzygj/s7652m/202003/a31191442e29474b98bfed5579d5af95.shtml (accessed March 4, 2020).

[B17] Nie X. D. Wang Q. Wang M. N. Zhao S. Liu L. Zhu Y. L. (2020). Anxiety and depression and its correlates in patients with coronavirus disease 2019 in Wuhan. *Int. J. Psychiatry Clin. Pract.* 1–6. 10.1080/13651501.2020.1791345. [Epub ahead of print]. 32662692

[B18] Pang S. X. (2016). Effect of nursing intervention on negative emotion and quality of life of patients with acute respiratory infection. *J. Qilu Nurs.* 22 32–34. 10.3969/j.issn.1006-7256.2016.09.017

[B19] Sharma A. Pillai D. R. Lu M. Doolan C. Leal J. Kim J. (2020). Impact of isolation precautions on quality of life: a meta-analysis. *J. Hosp. Infect.* 105 35–42. 10.1016/j.jhin.2020.02.004 32059996

[B20] Sheng Y. Gao F. L. Zhu L. X. (2003). Investigation and analysis for nursing needs of SARS patients with “functional health patterns” of Gordon. *Chin. J. Nurs.* 12 4–6.

[B21] Shi L. Lu Z. A. Que J. Y. Huang X. L. Liu L. Ran M. S. (2020). Prevalence of and risk factors associated with mental health symptoms among the general population in china during the coronavirus disease 2019 pandemic. *JAMA Netw. Open* 3:e2014053. 10.1001/jamanetworkopen.2020.14053 32609353 PMC7330717

[B22] Wang C. Horby P. W. Hayden F. G. Gao G. F. (2020). A novel coronavirus outbreak of global health concern. *Lancet* 395 470–473. 10.1016/S0140-6736(20)30185-9 31986257 PMC7135038

[B23] Wang Z. Y. Gao X. L. Cui Y. Y. Huang J. Y. Qian H. (2020). Analysis of anxiety and depression in patients with mild coronary pneumonia. *Psychologies* 15 40–42. 10.19738/j.cnki.psy.2020.20.014

[B24] Wei Z. X. Li Q. F. (2020). An overview of the clinical manifestations of novel coronavirus infection and its laboratory detection methods. *Int. J. Lab. Med.* 41 988–992. 10.3969/j.issn.1673-4130.2020.08.023

[B25] Weich S. Sloggett A. Lewis G. (1998). Social roles and gender difference in the prevalence of common mental disorders. *Br. J. Psychiatry* 173 489–493. 10.1192/bjp.173.6.489 9926077

[B26] World Health Organization (WHO) (2021). *World Health Organization Coronavirus Disease (COVID-19) Pandemic.* Avaliable online at: https://www.who.int/emergencies/diseases/novel-coronavirus-2019 (accessed April 30, 2021).

[B27] Xiao J. L. Chen Y. Fang F. Liu W. T. Zhong Y. Y. Tao J. (2020). Public anxiety and depression and its influencing factors under public health emergencies. *Modern Prev. Med.* 47 3557–3562.

[B28] Xiong J. Jiang W. L. Zhou Q. Hu X. Q. Liu C. Y. (2020). Clinical characteristics, treatment, and prognosis in 89 cases of COVID-19. *Med. J. Wuhan Univ.* 41 542–546. 10.14188/j.1671-8852.2020.0103

[B29] Xue Y. Z. Lu L. Liang Z. Q. Xu Y. Zhang K. R. (2008). Investigation on psychosomatic symptoms of SARS patients at different stages. *Chin. Prev. Med.* 04 268–270.

[B30] Yang H. (2004). *Exploration of Response of Psychology and Psychological Nursing Intervention in SARS Patients.* Jinzhong: Shanxi Medical University.

[B31] Zhang Y. (2012). Mental health needs survey and intervention analysis of infectious disease patients. *Med. Innov. China* 9 72–73. 10.3969/j.issn.1674-4985.2012.24.041

[B32] Zhang Y. Z. Li X. F. (2020). Investigation and analysis of current situation of pain management in inpatients. *Clin. Res. Pract.* 5 3–5. 10.19347/j.cnki.2096-1413.202010002

[B33] Zigmond A. S. Snaith R. P. (1983). The hospital anxiety and depression scale. *Acta Psych. Scand* 67 361–370. 10.1111/j.1600-0447.1983.tb09716.x 6880820

